# Isolated, but transnational: the *glocal* nature of Waldensian ethnobotany, Western Alps, NW Italy

**DOI:** 10.1186/s13002-015-0027-1

**Published:** 2015-05-07

**Authors:** Giada Bellia, Andrea Pieroni

**Affiliations:** Via del Pino 108, Pinerolo (Torino), I-10064 Italy; University of Gastronomic Sciences, Piazza Vittorio Emanuele 9, Bra/Pollenzo, I-12060 Italy

**Keywords:** Ethnobotany, Wild food plants, Medicinal plants, Alps, Italy

## Abstract

**Background:**

An ethnobotanical field study on the traditional uses of wild plants for food as well as medicinal and veterinary plants was conducted in four Waldensian valleys (Chisone, Germanasca, Angrogna, and Pellice) in the Western Alps, Piedmont, NW Italy. Waldensians represent a religious Protestant Christian minority that originated in France and spread around 1,170 AD to the Italian side of Western Alps, where, although persecuted for centuries, approximately 20,000 believers still survive today, increasingly mixing with their Catholic neighbours.

**Methods:**

Interviews with a total of 47 elderly informants, belonging to both Waldensian and Catholic religious groups, were undertaken in ten Western Alpine villages, using standard ethnobotanical methods.

**Results:**

The uses of 85 wild and semi-domesticated food folk taxa, 96 medicinal folk taxa, and 45 veterinary folk taxa were recorded. Comparison of the collected data within the two religious communities shows that Waldensians had, or have retained, a more extensive ethnobotanical knowledge, and that approximately only half of the wild food and medicinal plants are known and used by both communities. Moreover, this convergence is greater for the wild food plant domain. Comparison of the collected data with ethnobotanical surveys conducted at the end of the 19^th^ Century and the 1980s in one of studied valleys (Germanasca) shows that the majority of the plants recorded in the present study are used in the same or similar ways as they were decades ago. Idiosyncratic plant uses among Waldensians included both archaic uses, such as the fern *Botrychium lunaria* for skin problems, as well as uses that may be the result of local adaptions of Central and Northern European customs, including *Veronica allionii* and *V. officinalis* as recreational teas and *Cetraria islandica* in infusions to treat coughs.

**Conclusions:**

The great resilience of plant knowledge among Waldensians may be the result of the long isolation and history of marginalisation that this group has faced during the last few centuries, although their ethnobotany present trans-national elements.

Cross-cultural and ethno-historical approaches in ethnobotany may offer crucial data for understanding the trajectory of change of plant knowledge across time and space.

## Introduction

Ethnobotanical studies of minority and diasporic groups are of crucial interest in contemporary ethnobiology to help identify those cultural and/or social factors which affect the perceptions and uses of plants and to understand how traditional plant knowledge evolves [1-8].

Moreover, diverse analyses conducted in Europe during the last decade have pointed out that a broad range of factors influence the resilience of ethnobotanical knowledge and are able to slow or accelerate its erosion, including environmental changes, internal (urbanisation) and external migrations, self-perception and that of others’ identities, language, religion, as well as economic or political externalities [9-16].

On the other hand, the Alps have been shown to still represent an important reservoir of local, folk plant knowledge, both in touristic [17,18] and especially in “peripheral” valleys [19-22], which have been less affected by the mass tourism industry. 

Along these theoretical trajectories, our ethnobotanical research in recent years has focused on a number of linguistic “isles” and cultural boundaries in mountainous areas of Italy and the Balkans; especially in the latter cultural region, we have also observed the effect that religious affiliation has on the vertical transmission of folk plant knowledge, as it remarkably shapes kinship relations within multi-lingual and multi-religion communities [23].

In order to further assess the role that religion plays in shaping folk plant knowledge, we decided to investigate the local ethnobotany of the Waldensian community and that of their Catholic neighbours in the Western Alps, NW Italy. Waldensians represent a religious Christian (and later Protestant Christian) minority that originated in France during the 12^th^ Century which spread around 1,170 AD to the Italian side of the Cottian (Western) Alps. Harassed for centuries, Waldensians went through a long and dramatic history of persecutions, migrations and relocations, and, despite the isolation and marginalisation of their valleys, they built important ties to Protestant countries, notably England, the Netherlands, and Switzerland [24].

Nowadays, approximately 20,000 believers (Provençal/Occitan, Piedmontese and standard Italian speaking) still survive in these valleys, increasingly mixing with their Catholic neighbours.

The specific aims of this study were:

1. to record the local names and specific uses of wild food plants, as well as wild and non-wild plants for medicinal and veterinary practices in four Waldensians valleys;
2. to compare the ethnobotany of members belonging to the two faiths (Waldensians and Catholics); and
3. to diachronically compare the current data with those from the historical North Italian ethnobotanical data.

## Methods

### Selected sites

**Figure 1:**
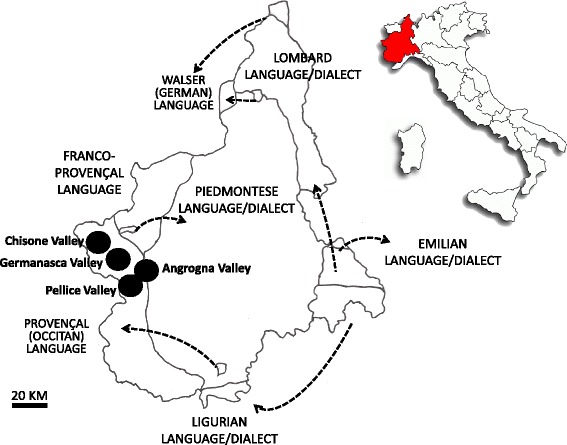
Location of the four considered Waldensian valleys within the linguistic map of Piedmont, NW Italy.

Figure 1 shows the location of the study sites, which were represented by four Waldensian valleys (Chisone, Germanasca, Angrogna, and Pellice) located in the Western Alps, Piedmont, NW Italy.

The valleys are characterized by chestnut (*Castanea sativa* Mill.), beech (*Fagus sylvatica* L.), and larch (*Larix decidua* Mill.) forests, with some Scots pine (*Pinus sylvatica* L.); the climate is alpine, with relevant annual precipitations (1000–2000 mm/year).

In particular, the following villages were visited: Fenestrelle (1,138 m.a.s.l.), Mentoulles (1,046 m.a.s.l.), Villaretto (986 m.a.s.l.), Pomaretto (619 m.a.s.l.), Campo La Salza (1,140 m.a.s.l.), Massello (1,187 m.a.s.l.), San Martino (1,063 m.a.s.l.), Villasecca (832 m.a.s.l.), Angrogna (582 m.a.s.l.), and Bobbio Pelice (762 m.a.s.l.).

All villages officially report a few hundred inhabitants (normally 300–500), but the actual figures are largely overestimated, as a significant portion of the current resident populations lives in the lowland Piedmontese centres and Turin and comes back to the villages only during the summer or on the weekends.

The local economy, since a few decades, is no longer based on agro-pastoral activities, and the elderly inhabitants live off of their pensions and in their free time manage some home-gardens and/or small-scale agricultural activities. Young and mid generations work instead in the main lowlands centres and in Turin.

Mass tourism is absent, although some eco-touristic initiatives have been growing in recent years.

The original Waldensian inhabitants have increasingly mixed with their Catholic neighbours in the last few decades, and in most cases intermarriage leads to a family’s change of faith (from Waldensian to Catholic).

Nowadays the language spoken within the domestic arena is increasingly a mixture of the original Provençal/Occitan language with the Piedmontese variety of Italian. All inhabitants also speak standard Italian.

### Field study

In the years 2010–2014, forty-seven elderly informants (nineteen Catholics and twenty-eight Waldensians, aged between 58 and 78 years) were selected, among those locals who could be identified as Traditional Knowledge holders (normally elderly small-scale farmers and shepherds), employing snowball sampling techniques. These individuals then were interviewed after Prior Informed Consent was verbally obtained.

The focus of the interviews, which were conducted in standard Italian, was the folk knowledge (name and use) of wild food plants and wild and non-wild medicinal and veterinary plants.

The Code of Ethics of the International Society of Ethnobiology [25] was strictly followed.

The wild plant species mentioned by the informants were collected, when available, identified according to Flora d’Italia [26], and finally stored at the Herbarium of the University of Gastronomic Sciences.

Plant family assignments follow the current Angiosperm Phylogeny Group designations [27].

The reported folk plant names were transcribed using the rules of the Provençal/Occitan and standard Italian languages.

### Data analysis

We compared the data gathered among local Waldensians with those collected among Catholics in the same study sites.

Moreover, we compared our findings with those observed in two ethnobotanical field studies conducted in the same areas (Val Germanasca) at the end of the 19^th^ Century and in the 1980s [28-30]. In particular, the first work represents one of the very first ethnobotanical studies in Italy as well as the whole of Europe, which was conducted by a Waldensian botanist working as a secondary school teacher, who died from an infectious disease in Uruguay, where he immigrated one year after the publication of his investigation [31].

## Results and discussion

### Wild food plants

Table 1 shows the recorded uses of the wild food and semi-domesticated plant taxa.

**Table 1:** **Local wild or semi-domesticated food plant uses recorded in the studied area**

**Botanical taxon/family and voucher specimen code**	**Recorded local names**	**Plant part(s)**	**Local culinary use(s)**	**Wal**	**Cat**	**Citations**	**Notes**
*Achillea erba-rotta* All. Asteraceae UNISGVALACH	Routto Ruta di montagna	Aerial parts	Home-made liqueurs	+		*	C
*Achillea millefolium* L. Asteraceae UNISGVGB025	Primmoflour	Leaves	Soups		+	*	P
*Alchemilla xanthochlora*Rothm. Rosacea UNISGVGB030		Leaves	Soups	+	+	*	P
*Allium schoenoprasum* L. Amaryllidaceae UNISGVALALL	Aiet	Leaves	Seasoning (salads)	+		*	C
*Allium ursinum* L. Amaryllidaceae UNISGVALALU		Leaves	Ingredients for soups	+	+	*	P
*Amelanchier ovalis* Medik. Rosaceae	Amarenchie	Fruits	Eaten raw		+	*	P
*Angelica sylvestris* L. Apiaceae UNISGVGB002	Angelica	Roots	Home-made liqueurs	+		*	C
*Anthriscus sylvestris* (L.) Hoffm. Apiaceae UNISGVALANT	Chafoulhét	Leaves	Salads	+		*	P
*Arctostaphylos uva-ursi* (L.) Spreng. Ericaceae UNISGVALARC	Pan dë vouëlp Pinmerlés	Fruits	Jams	+	+	*	P
*Arctium lappa* L. Asteraceae UNISGVGB034	Grattëquioùe	Very young leaves	Soups		+	*	P
*Artemisia genipi* Weber ex. Stechm., *A.glacialis* L., *A. umbelliformis* Lam*.* Asteraceae UNISGVALAGE UNISGVALAGL UNISGVALARU	Genepì Gënëpi fumél (*A. umbelliformis*)Gënëpi macle (*A.genipi*)	Flowering tops	Home-made liqueurs	+		*	C
*Artemisia vulgaris* L. Asteraceae UNISGVGB038	Arsemizë Eisente Ërsëmizo	Leaves	Seasoning soups or omelettes	+		*	C
*Aruncus dioicus* (W.)F. Rosaceae UNISGVGB040	Glaudia	Shoots	Boiled	+	+	**	P
*Asparagus tenuifolius* Lam. Asparagaceae UNISGVALASP	Aspèrge selvagge	Shoots	Boiled	+	+	*	P
*Bellis perennis* L. Asteraceae UNISGWAL007	Magritin Margaritin	Leaves and flowers	Salads, soups, omelettes, risotto	+		*	C
*Berberis vulgaris* L. Berberidaceae UNISGVALBER	Pittou	Fruits	Jams	+	+	**	P
*Beta vulgaris* L. Amaranthaceae	Bléo	Leaves	Cooked	+		*	C
*Borago officinalis* L. Boraginaceae UNISGWAL013	Bouràes Bourai Burài	Leaves and flowers	Soups, salads, omelettes	+	+	***	C
*Campanula rapunculus* L. Campanulaceae UNIGVALCAM	Rampoun	Leaves and roots	Salads	+		*	C
*Capsella bursa- pastoris* (L.) Medik. Brassicaceae UNISGVALCAP		Young leaves	Omelettes	+		*	P
*Carlina acaulis* L. Asteraceae UNISGVALCAR	Chardouso	Flowers	Macerated in olive oil; the resulting oil used as seasoning	+	+	*	C
*Carum carvi* L. Apiaceae UNISGVALCAU	Chiréi Cummel	Fruits	Seasoning, home-made liqueurs	+	+	**	C
*Centaurea scabiosa* L. Asteraceae UNISGVALCEN		Young leaves	Soups	+	+	*	P
*Cerinthe* sp. (?) Boraginaceae	Anhaoù grò	Leaves	Boiled		+	*	P
*Chenopodium album* L. Amaranthaceae UNISGVALCHE	Sënicle	Leaves	Soups, boiled, omelettes	+	+	*	P
*Chenopodium bonus-henricus* L. Amaranthaceae UNISGWAL017	Orla Parch	Leaves	Soups, omelettes, boiled	+	+	***	C
*Cichorium intybus* L. Asteraceae UNISGVALCIC	Sicorio	Young leaves Roots	Salads Roasted and grounds as coffee substitute	+	+	*	C P
*Corylus avellana* L. Betulaceae UNISGVALCOR		Seeds	Consumed raw		+	*	P
*Daucus carota* L. Apiaceae UNISGVALDAU	Carotto	Roots	Salads	+		*	C
*Dryas octopetala* L. Rosaceae UNISGVALDRY		Leaves and flowers	Cosumed raw as a snack	+		*	P
*Fragaria vesca* L. Rosaceae UNISGWAL036	Maiùssa	Leaves Fruits	Soups, salads Jams	+	+	**	C
*Gentiana acaulis* L. Gentianaceae UNISGVGB027	Braio d’cucuc Pirulet	Roots, flowers	Home-made liqueurs	+	+	**	C
*Gentiana lutea* L. Gentianaceae UNISVALGEN	Argensiana Gënsano	Roots	Home-made liqueurs (or wine macerates)	+	+	***	C
*Humulus lupulus* L. Cannabaceae UNISGWAL015	Lüvërtin Luvertìn	Shoots	Omelettes, boiled	+	+	***	C
*Juniperus communis* L. Cupressaceae UNISVALJUN	Gënébbre	Galbules	Seasoning	+	+	***	C
*Lapsana communis* L. Asteraceae UNISGVALLAP	Jalino graso	Young leaves	Soups, omelettes, boiled	+	+	***	C
*Laurus nobilis* L. Lauraceae UNISGVALLAU	Loriè	Leaves	Seasoning	+		*	C
*Leontodon hispidus* L. (?) (Asteraceae)	Plissa	Leaves	Salads, soups	+		*	P
*Leontopodium nivale* (Ten.) Huet ex Hand.-Mazz. Asteraceae UNISGVALLEO	Stela alpina	Flowering tops	Home-made liqueurs	+		*	C
*Lonicera caerulea* L. Caprifoliaceae UNISGVALLON	Èrza d’loup	Flowers	Eaten raw as a snack	+		*	P
*Malva sylvestris* L. Malvaceae UNISGVAMAL	Màevë Malvo	Leaves	Soups	+	+	*	C
*Mentha longifolia* (L.) L. Lamiaceae UNISGVALMEN	Mëntatre	Leaves	Seasoning (esp. soups and omelettes)	+		*	P
*Nasturtium officinale* R.Br. Brassicaceae UNISGVALNAS	Creisoun	Leaves	Salads	+	+	***	C
*Origanum vulgare* L. Lamiaceae UNISGVALORI	Origano	Leaves	Seasoning	+		*	C
*Oxalis acetosella* L. Oxalidaceae UNISGVALOXA	Èrbo dâ cucuc Pan d’ûzèl	Leaves	Salads		+	*	C
*Parietaria officinalis* L.Urticaceae UNISGVGB007	Pan-chaoudét	Leaves	Soups	+		*	P
*Pedicularis foliosa* L. Orobanchaceae UNISVALPED		Flowers	Sucked as a snack (by children)		+	*	P
*Persicaria bistorta* L. Polygonaceae UNISGVALPER	Albubuine Arparô Ërparâ	Young leaves	Soups	+	+	**	C
*Pinus cembra* L. Pinaceae UNISGVALPCE	Èlvou	Seeds	Consumed raw		+	**	P
*Pinus sylvestris* L. Pinaceae UNISGVALPSY	Pin	Seeds	Consumed raw		+	*	P
*Plantago major* L. Plantaginaceae UNISGVGB021	Plantanh Plantònh	Leaves	Soups	+	+	**	P
*Physalis alkekengi* L. Solanaceae UNISGWAL040	Erba chiocca Fiacch Puvron selvaj	Fruits	Jams	+		*	P
*Phyteuma spicatum* L. Campanulaceae UNISGWAL043	Iucca	Young leaves and shoots	Soups	+		*	P
*Polypodium vulgare* L. Polypodiaceae UNISGVGB003	Ërgalisio Rizouzèttë	Roots	Consumed raw as a snack and as a seasoning for home-made beverages	+	+	***	C
*Portulaca oleracea* L. Portulacaceae UNISGVALPOR	Pouslano	Young leaves (before flowering)	Salads	+		*	P
*Primula helatior* (L.) Hill, *P. veris* L., *P. vulgaris* Huds. Primulaceae UNISGVALPRE UNISGVALPVEUNISGVALPVU	Coucouc Pimpette Pimpinéllo	Young leaves and flowers	Salads, soups, omelettes	+	+	***	C
*Prunus avium* (L.) L. Rosaceae UNISGVALPRA	Sireizie	Fruits	Consumed raw or in jams		+	*	P
*Prunus brigantina* Vill. Rosaceae UNISGVALPRB	Marmouti	Fruits	Consumed raw or in jams	+	+	*	P
*Prunus spinosa* L. Rosaceae UNISGVALPRS	Agrenié Bousou niër	Fruits	Jams	+	+	**	P
*Ribes alpinum* L. Grossulariaceae UNISGWAL023	Uopastrìe	Fruits	Consumed raw or in jams	+	+	**	P
*Ribes uva-crispa* L. Grossulariaceae UNISGVALRUC	Groouzèlla	Fruits	Consumed raw or in jams		+	*	P
*Robinia pseudoacacia* L. Fabaceae UNISGVALROB	Gazhillo	Flowers	Deep-fried (in batter)	+		*	C
*Rosa canina* L. Rosaceae UNISGVGB018	Agoulensië Bosou	Fruits	Jams	+	+	***	C
*Rubus ulmifolius* L. Rosaceae UNISGWAL038	Rounzo	Young leaves Fruits	Soups Jams	+		*	P
*Rubus idaeus* L. Rosaceae UNISGWAL037	Ampolen Ampoulie	Fruits	Jams, syrups	+	+	**	C
*Rumex acetosa* L. Polygonaceae UNISGVGB011	Aseuccla Asuitta di pra Isìgula Situla	Stems Leaves	Consumed raw as a snack (stems); salads, soups, omelettes, boiled	+	+	***	C
*Rumex alpinus* L. Polygonaceae	Lapòs Lavasa Rabarbaro selvatico	Stem Leaves	Jams Soups	+	+	**	P
*Salvia pratensis* L. Lamiaceae UNISGVGB033	Bounom	Young leaves	Soups	+	+	**	P
*Sambucus nigra* L. and *S. racemosa* L. Adoxaceae UNISGWAL016 *(S. nigra)*	Sèuc, Seuic	Flowers Fruits	Deep fried (in batter) or seasoning home-made beverages Jams	+	+	***	C
*Silene vulgaris* (Moench.) Garcke Caryophyllaceae UNISGVGB20	Chersinet Cresinet Eicloupèt	Young leaves	Soups, omelettes, boiled	+	+	***	C
*Tanacetum vulgare* L. Asteraceae UNISGWAL009	Archebüse Tanaìa Tanaìo	Leaves	Seasoning soups (esp. a local bread-based soup [*suppa barbetta*]), home-made liqueurs, omelettes	+	+	***	C
*Taraxacum officinale* (L.) Weber Asteraceae UNISGWAL010	Girasole Mourpoursin	Leaves Roots Flower heads	Salads, soups Roasted and grinded as a substitute of coffee Pickled in brine and used as flavouring	+	+	***	C
*Thymus serpyllum* L. Lamiaceae UNISGWAL029	Serpoul	Flowers and leaves	Seasoning (also for cheese and a local bread-based soup [*suppa barbetta*]), home-made liquors	+	+	***	C
*Tragopogon pratensis* L. Asteraceae UNISGWAL011	Barbabouc	Young leaves	Soups, omelettes, boiled	+	+	***	C
*Trifolium* spp. Fabaceae	Fioun	Flowers	Deep fried (in batter)	+		*	P
*Tussilago farfara* L. Asteraceae UNISGVALTUS	Pimpetta Ounglëtto	Young leaves	Salads		+	*	P
*Urtica dioica* L. Urticaceae UNISGWAL041	Urtìa Urtìo Ürtia	Leaves	Soups, omelettes, risotto	+	+	***	C
*Vaccinium myrtillus* L. Ericaceae UNISGVALVAM	Ërzaìe Èidra	Fruits	Jams, syrups	+	+	***	C
*Vaccinium vitis-idaea* L. Ericaceae UNISGVALVAV	Panféino	Fruits	Jams	+		*	P
*Valerianella locusta* (L.) Laterr. Caprifoliaceae UNISGVALVAL	Saladét	Leaves	Salads	+	+	**	P
*Veronica allionii* Vill. Plantaginaceae UNISGVALVEA	Èrbë d’tè GiaspertereTé d’mountannho	Leaves and flowers	Recreational tea	+	+	*	P
*Veronica officinalis* L. Plantaginaceae UNISGVALVEO	Èrbë d’tè Tè svizzero	Leaves and flowers	Recreational tea	+		*	C
*Viburnum lantana* L. Adoxaceae UNISGVALVIB	Tatoulie	Fruits	Consumed raw		+	*	P
*Viola tricolor* L. Violaceae UNISGVGB005	Violette Viooulëtìn Vioulëtto blancho	Leaves and flowers	Salads, soups	+	+	**	C
*Unidentified taxon*	Sparsi	Leaves and flowers	Salads, soups, omelettes	+		*	P

(?) identification only via plant and habitat descriptions and folk names.

Wal: use recorded among Waldensians; Cat: use recorded among Catholics.

Notes: C: current use; P: past use.

Citations: *quoted by 10% of the informants or less; **quoted by 11-39% of the informants; ***quoted by 40% of the informants or more.

The collection of the young aerial parts of the following wild vegetables is still common in the study area: *Borago officinalis*, *Primula* spp., *Nasturtium officinale, Lapsana communis, Chenopodium bonus-henricus, Rumex acetosa, Tragopogon pratensis, Urtica dioica, Silene vulgaris, Humulus lupulus*, and *Taraxacum officinale.*

The above confirms what we already know about wild food plant consumption in Italy and in particular NW Italy, where the very common consumption of the young shoots of *Humulus lupulus* and *Tragopogon pratensis* can be considered a cultural marker of Piedmontese cuisine. While all these data confirm the observations reported nearly one century ago by Giovanni Mattirolo in his review of the wild plants of Piedmont [32], it appears that the practice of gathering and consuming the leaves/young shoots of *Valerianella locusta, Phyteuma* spp., *Persicaria bistorta*, and *Aruncus dioicus* continued only until the recent past and/or is less common today. The latter three species (in soups or boiled) in particular represent an important part of the slowly disappearing North Italian Alpine culinary “traditions” [17,33].

**Figure 2:**
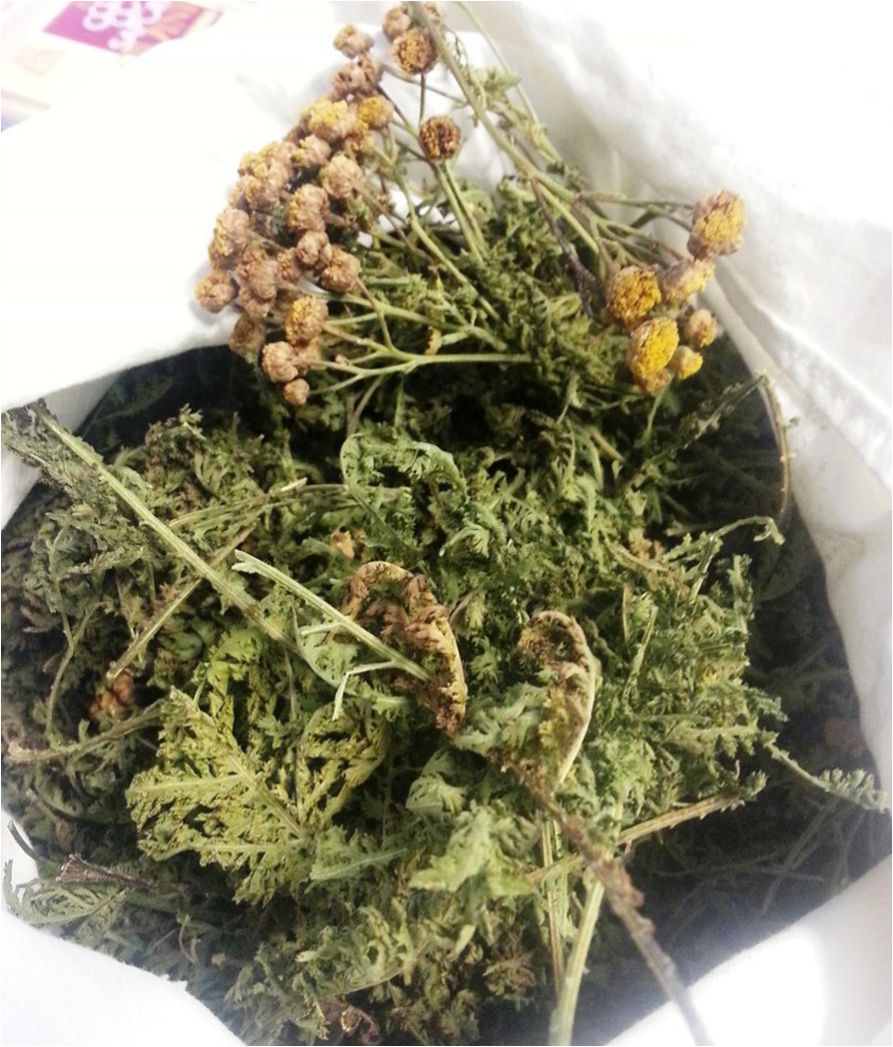
Dried aerial parts and flowers of *Tanacetum vulgare.*

Among the wild plants exploited for seasoning, the use of *Carum carvi, Thymus serpyllum, Juniperus communis*, and *Tanacetum vulgare* is predominant. In particular, the common use of the leaves of the last species (Figure 2) – which has been widely reported not only in the Piedmont region but also recently in Occitan/Provençal and Alpine Ligurian areas [17,22,34,35] – as a crucial seasoning ingredient in omelettes, soups, and a home-made liqueur called *arquebuse* may be better investigated from a historical perspective. In fact, this species has a long history of folk use in Britain, especially in omelettes consumed during the fish-based diet of Lent [36], and Waldensians, even in the poorest villages, have maintained for many centuries intense cultural ties to Britain, due to the historical and theological proximity between the Protestant/Anglican and Waldensian faiths [23].

**Figure 3:**
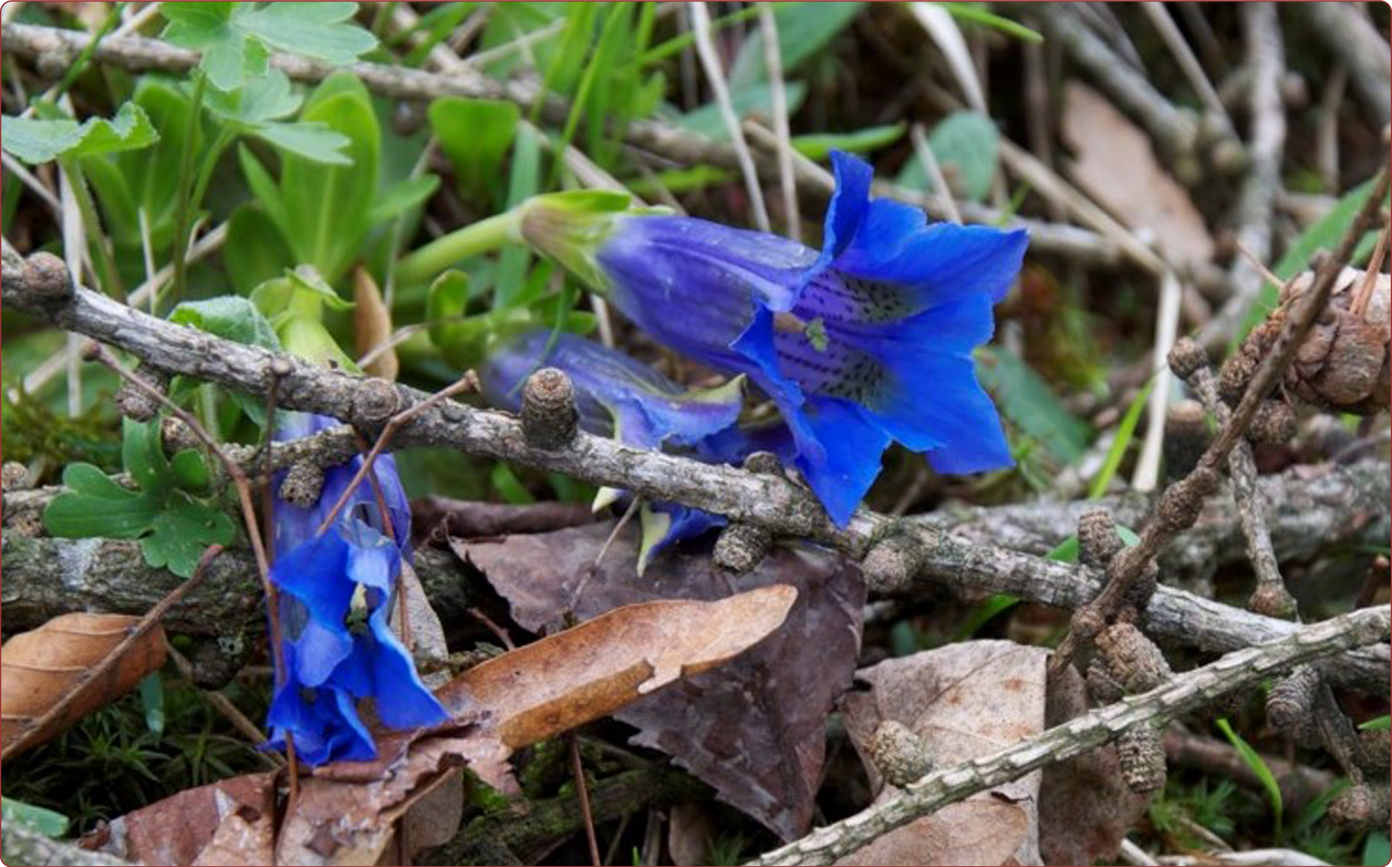
Gentiana acaulis.

As in other areas of NW Italy ([17], and references therein), wild *Artemisia genipi, A. glacialis,* and *A. umbelliformis* flowering tops (*genepì*), *Gentiana acaulis* flowers (Figure 3) and roots, and *G. lutea* roots are commonly gathered and used for making home-made hydro-alcoholic macerates/digestive liqueurs.

Among wild fruits, the gathering of the fruits/pseudo-fruits of *Rosa canina, Sambucus nigra* (and rarely *S. racemosa*), and *Vaccinium myrtillus* is still commonly practiced.

Finally, the frequent use of the aerial parts of *Veronica* species (esp. the local *Veronica allionii*) as recreational teas in the study area, which has also been recorded in adjacent valleys [17], could be the result of cultural “contamination” from British and Northern/Central European customs. Waldensians, for example, have introduced in their valleys, and continue to practice today, the English custom of taking afternoon tea, which is extremely uncommon among the autochthonous Catholics in the study area as well as other areas of Italy.

In place of exotic and expensive colonial teas, the poor villagers may have opted for a “cheap”, local substitute, which may explain the use of the aerial parts of *Veronica* spp. even today. This tea – sometimes locally and more recently called “Occitan tea” - became in the last decade in the study area and also among the entire Occitan/Provençal community living in the Western Italian Alps an important cultural marker and seems to represent there one of the distinctive signs of the local identity.

On the other hand, the use of *Veronica officinalis* tea was very spread in France, Switzerland, and Northern Europe in the 19^th^ Century [37].

### Medicinal plants

Table 2 reports the locally recorded medicinal plant uses.

**Table 2:** **Local medicinal plant uses recorded in the studied area**

**Botanical taxon/ family and voucher specimen code #**	**Local names**	**Status**	**Plant parts**	**Preparation and administration**	**Folk medical use(s) or treated disease(s)**	**Wal**	**Cat**	**Citations**	**Notes**
*Abies alba* Mill. Pinaceae UNISGVALABA	Bigiun Sap Sòp blanc	W	Buds Resin	Infusion, syrup Topical application	Cough Skin infections, arthritis, bruises	+	+	***	C
*Acer pseudoplatanus* L. Sapindaceae UNISGVALACE	Plai Plaie	W	Leaves	Infusion	Cough, flu		+	*	P
*Achillea erba-rotta* All. Asteraceae	See Table 1	W	Aerial parts	Infusion, liquor	Digestive, fever	+	+	*	C
*Alchemilla xanthochlora* Rothm. Rosaceae		W	Aerial parts	Infusion Topical application	Anti-inflammatory Dysmenorrhea	+	+	**	P
*Allium ampeloprasum* L. Amaryllidaceae	Pourëtto	C	Roots	Decoction	To decrease the milk secretion	+		*	P
*Allium sativum* L. Amaryllidaceae	Alh	C	Bulb	Topical application Fumigations Externally applied to cloths	Corns Cough Worms	+	+	*	P
*Aloysia citriodora* Palau Verbenaceae	Limonella	C	Leaves	Infusion	Dysmenorrhea	+		*	C
*Arctium lappa* L. Asteraceae UNISGVGB034	Grattëquioùe	W	Roots Flowers	Decoction Infusion	Respiratory infections, fever, “blood thinner”	+	+	**	P
*Arctostaphylos uva-ursi* (L.) Spreng. Ericaceae	See Table 1	W	Leaves	Infusion	Diuretic and inflammations of the urinary tract	+		*	P
*Arnica montana* L. Asteraceae UNISGWAL003	Tabacas Tabaccai	W	Flowers	Tincture or macerate in olive oil, externally applied	Rheumatisms, arthritis muscle pains, bruises	+	+	***	C
*Artemisia absinthium* L. Asteraceae UNISGWAL004	Ûsenc	W	Aerial parts	Topical application Infusion	Bruises Fever, worms, digestive	+	+	***	P
*Artemisia genipi* Weber ex. Stechm., *A.glacialis* L., *A. umbelliformis* Lam*.* Asteraceae	See Table 1	W	Aerial parts	Liquor, infusion	Digestive, cough	+	+	***	C
*Artemisia vulgaris* L. Asteraceae UNISGVGB038	Arsemizë Ërsëmizo	W	Aerial parts	Infusion Topical application	Dysmenorrhea Bruises	+	+	**	P
*Beta vulgaris* L. Amaranthaceae	Bléo	C	Leaves	Topical application	Joint pains, acne	+		*	P
*Borago officinalis* L. Boraginaceae UNISGWAL013	Bouràes Bourai	C	Flowers	Infusion Topical application	Pimples Eczema, psoriasis	+		**	P
*Brassica oleracea* L. Brassicaceae	Chôl	C	Leaves	Topical application	Pimples, acne	+	+	**	C
*Botrychium lunaria* (L.) Sw. Ophioglossaceae UNISGVALBOT	Èrbo d’l’uo	W	Ripe sporangium	Topical application Inhalation Infusion	Skin wounds Nose bleeding Internal bleeding	+		**	P
*Brassica rapa* L. Brassicaceae	Rabbo	C	Bulb	Syrup	Cough	+		*	C
*Calendula officinalis* L. Asteraceae	Courtëzio	C	Flowers	Infusion	Dysmenorrhea, for promoting blood circulation	+		**	C
*Capsella bursa-pastoris* (L.) Medik. Brassicaceae		W	Fruits	Topical application	Skin wounds	+		*	P
*Carum carvi* L. Apiaceae	See Table 1	W	Fruits	Infusion, liquor	Digestive, carminative	+	+	*	C
*Cetraria islandica* L.(Ach.) Parmeliaceae UNISGVALCET	Èrbo d’la vélho Licchia Lichene Pan d’chabbre	W	Thallus	Decoction, syrup Decoction, externally applied	Cough, bronchitis	+	+	***	C
*Chelidonium majus* L. Papaveraceae UNISGVGB039	Sireunnho Erbë sironnhë	W	Latex	Fresh topical applied	Warts	+	+	***	C
*Conium maculatum* L. Apiaceae UNISGVALCON	Sicutto	W	Aerial parts	Infusion	Abortive	+		*	P
*Crataegus monogyna* Jacq. Rosaceae UNISGVALCRA	Prusét	W	Aerial parts	Infusion	Hypertensive, venous insufficiency	+	+	**	P
*Cyanus segetum* Hill. Asteraceae UNISGVGB015		W	Flowers	Eyebaths	Conjunctivitis	+		*	P
*Cynodon dactylon* L. (Pers.) Poaceae UNISVALCYN	Gramoun	W	Roots	Decoction	Diuretic	+	+	*	P
*Datura stramonium* L. Solanaceae UNISGVALDAT	Èrbo dâ dërboun	W	Leaves	Inhalation (dried powedered leaves)	Asthma	+		*	P
*Equisetum arvense* L. Equisetaceae UNISGWAL020	Èrbo cavalino	W	Sterile stem	Decoction Topical application	Diuretic, to prevent prostatic cancer Skin inflammations	+	+	***	C
*Erica carnea* L. Ericaceae UNISGVALERI	Erica	W	Aerial parts	Infusion	Urinary tract infections, diarrhea	+		*	P
*Euphrasia alpina* Lam. Orobanchaceae UNISGVALEUP	Eufrasia	W	Flowers	Eyebaths	Conjunctivitis	+		*	P
*Fraxinus excelsior* L. Oleaceae UNISGVGB022	Fraise	W	Leaves	Infusion	Venous insufficiency, hypertension	+		*	P
*Fragaria vesca* L. Rosaceae	See Table 1	W	Leaves	Topical application	Pimples, acne	+		*	P
*Gentiana acaulis* L. Gentianaceae	See Table 1	W	Whole plant	Liquor, infusion	Apetizing, digestive	+	+	**	P
*Gentiana lutea* L. Gentianaceae	See Table 1	W	Roots	Liquor Macerated in wine	Appetizing, digestive	+	+	***	C
*Hypericum perforatum* L. Hypericaceae UNISGWAL018	Millepertuis Trafourèllo Sengian	W	Flowering aerial parts	Macerate in oil	Skin inflammations, burnes, arthritis	+	+	***	C
*Hyssopus officinalis* L. Lamiaceae	Izòp	C	Aerial parts	Infusion	Cough	+		*	P
*Juglans regia* L. Juglandaceae	Nouvìe	C	Leaves	Infusion, externally applied	Chilblains	+	+	*	P
*Juniperus communis* L. Cupressaceae	See Table 1	W	Fruits	Infusion, liquor	Digestive	+	+	*	C
*Lamium album* L. Lamiaceae UNISGVALLAM	Urtìo morto	W	Aerial parts	Infusion	Dysmenorrhea	+		*	P
*Larix decidua* Mill. Pinaceae UNISGVGB031	Mèlze	W	Sprouts Resin Pine cones	Infusion Topical application Syrup	Expectorant Skin inflammations (remove splinters) Respiratory infections	+	+	**	C
*Laurus nobilis* L. Lauraceae	See Table 1	W	Leaves Fruits	Infusion	Digestive		+	*	P
*Leontopodium nivale* (Ten.) Huet ex Hand.-Mazz. Asteraceae	See Table 1	W	Flowering tops	Infusion	Digestive	+		*	C
*Linum usitatissimum* L. Linaceae	Lin	C	Seeds	Poultice, externally applied Baths Macerated in water	Respiratory infections Urinary infections, constipation Toothaches	+	+	**	P
*Malva sylvestris* L. Malvaceae UNISGWAL031	Malvo	W	Whole plant	Decoctions	Urinary and genital tracts inflammations, digestive	+	+	***	C
*Marrubium vulgare* L. Lamiaceae UNISGVALMAR	Marëfi	W	Whole plant	Infusion	Digestive	+		*	P
*Matricaria chamomilla* L. Asteraceae UNISGWAL008	Caramillho	W	Flowers	Infusion Infusion, externally applied in poultices Oleolites	Urinary tract infections Bronchitis Earaches	+	+	***	C
*Melissa officinalis* L. Lamiaceae UNISGWAL026	Melissa	C	Leaves	Infusion	Neurorelaxant	+	+	**	C
*Menta longifolia* (L.) Huds. Lamiaceae	Mëntatre	W	Leaves	Infusion	Digestive	+	+	*	C
*Myristica fragrans* Houtt. Myristicaceae	Noce moscata	C	Seeds	Grinded and ingested with sugar	Dysmenorrhea	+		*	P
*Ononis spinosa* L. Fabaceae	Ratabuou	W	Roots	Decoction	Cystitis, in the prevention of prostate cancer	+		*	P
*Origanum vulgare* L. Lamiaceae	Oouriënt	C	Leaves	Infusion	Digestive	+		*	C
*Parietaria officinalis* L. Urticaceae UNISGVGB007	Pan-chaoudét	W	Aerial parts	Infusion	Urinary tract infections and for prevention prostate cancer	+	+	***	C
*Pelargonium zonale* (L.) L’Hér. ex Aiton Geraniaceae	Geranio odoroso	C	Leaves	Topically applied (fresh)	Skin cuts, hamatomas, wounds	+		**	P
*Pilosella officinarum* Vaill. Asteraceae UNISGVGB013	Èrbo dâ runh Ourèllhë d’rattë	W	Leaves	Topical ly applied (fresh)	Skin cuts and wounds		+	*	P
*Pinguicola vulgaris* L. Lentibulariaceae	Èrbo d’la talheuiro	W	Leaves	Topically applied (fresh)	Skin cuts, wounds	+		**	P
*Pinus cembra* L. Pinaceae	Èlvou	W	Cones Resin	Syrup Topically applied	Expectorant Wounds		+	*	C
*Pinus mugo* Turra, *P. sylvestris*	Pin	W	Cones, Sprouts	Syrup, Decoction	Cough, bronchitis	+	+	**	P
L. Pinaceae									
*Plantago major* L., *P. lanceolata* L*.* Plantaginaceae UNISGVGB021	Plantanh Plantònh	W	Leaves	Infusion Baths Topically applied (fresh)	Urinary and genital infections To prevent prostate cancer Bruises and haematomas	+	+	**	P
*Polygonum bistorta* L. Polygonaceae UNISGVGB036	Ërparà	W	Aerial parts	Infusion	Diuretic		+	*	P
*Polypodium vulgare* L. Polypodiaceae UNISGVGB003	Ërgalisio Rizouzèttë	W	Roots	Decoction	Cough, digestive	+	+	**	P
*Potentilla reptans* L. Rosaceae UNISGVALPOT	Èrbo d’la sinquèno	W	Whole plant	Decoctions Baths	Urinary infections To prevent prostate cancer	+	+	*	P
*Primula helatior* (L.) Hill, *P. veris* L., *P. vulgaris* Huds. Primulaceae	See Table 1	W	Flowers and roots	Infuson/Decoction	Diuretic, cough		+	*	P
*Prunus avium* (L.) L.	See Table 1	W	Stems Resin	Infusion Topically applied	Diuretic Sprains	+	+	**	P
*Prunus domestica* L. Rosaceae	Dalmeizinìe	C	Resin	Topically applied	Skin cuts and sprains	+		*	P
*Prunus dulcis* (Mill.) D.A. Webb Rosaceae	Amandoulie	C	Seeds	Fresh eaten	Galactagogue	+		*	P
*Rhododendron ferrugineum* L. Ericaceae UNISGVGB035	Brousé	W	Galls	Oleolite	Muscle pains	+		*	C
*Rosa canina* L. Rosaceae UNISGVGB018	Bosou Agoulënsia	W	Fruits Flowers	Jam Decoction Infusion, in external applications on the eyes	Intestinal astringent Increase immunostimulating Eye inflammations and conjunctivitis	+	+	**	P
*Rosa centifolia* L. Rosaceae	Ruse	C	Petals	Infusion	Sore throat	+		*	C
*Rosmarinus officinalis* L. Lamiaceae UNISGWAL030	Rousmarin	C	Leaves	Infusion	Digestive	+	+	*	C
*Rubus ulmifolius* L. Rosaceae UNISGWAL038	Rounzo	W	Leaves	Infusion Topical fresh applied	Sore throat and hoarseness Acne and pimples, cicatrizing	+	+	**	P
*Rumex acetosa* L. Polygonaceae	See Table 1	W	Leaves	Topically applied (fresh)	Insect bites	+		*	P
*Rumex alpinus* L. Polygonaceae	Lavaso	W	Leaves	Infusion	Cough	+		*	P
*Salix alba* L. Salicaceae	Gourìe	W	Leaves	Infusion	Fever	+		*	P
*Salvia officinalis* L. Lamiaceae	Salvio	C	Leaves	Infusion	Oral disinfectant and antibacterial, headaches, digestive	+	+	**	C
*Sambucus nigra* L. Adoxaceae	See Table 1	W	Flowers Fruits	Infusion Applied (fresh) in the mouth Jam	Hypertension Tooth abscess “Blood cleanser”	+	+	**	P
*Satureja montana* L. Lamiaceae	Sëréa	W	Flowers	Infusion	Dysmenorrhea		+	*	P
*Sempervivum montanum* L. Crassulaceae UNISGVGB029		W	Aerial parts	Topically applied (fresh)	Skin cuts and burns		+	*	P
*Silybum marianum* (L.) Gaertn. Asteraceae UNISGVALSYL	Pugn	W	Leaves Roots	Infusion Decoction	Diuretic, dysmenorrhea	+		*	P
*Symphytum officinale* L. Boraginaceae UNISGVALSYM	Èrbo dâ panariss	W	Roots	Topicaly applied (fresh)	Muscle pains and skin infections	+		*	P
*Tanacetum vulgare* L. Asteraceae UNISGWAL006	Tanaìo	W	Aerial parts	Infusion	Dysmenorrhea	+		*	P
*Taraxacum officinale* L. Asteraceae UNISGWAL010	Girasole Mourpoursin	W	Roots	Decoction	Diuretic/“blood cleasing”	+	+	*	P
*Teucrium chamaedrys* L. Lamiaceae UNISGVGB019	Calamandréo	W	Aerial parts	Infusion	Hypertension, dysmenorrhea	+	+	**	P
*Thymus serpyllum* L. Lamiaceae UNISGWAL029	Sërpoul	W	Aerial parts	Infusion Topically applied (fresh)	Digestive Insect bites	+	+	***	C
*Tilia cordata* Mill. Malvaceae UNISGVALTIL	Télh Tîeul	W	Flowers	Infusion	Respiratory tract inflammations	+	+	***	C
*Trigonella caerulea* (L.) Ser.	Thé d’hl’ort	C	Aerial parts	Infusion	Digestive	+		*	C
Fabaceae									
*Tussilago farfara* L. Asteraceae	See Table 1	W	Aerial parts	Infusion	Respiratory tract inflammations, fever		+	**	P
*Urtica dioica* L. Urticaceae UNISGWAL031	Urtìo	W	Roots	Decoction	Diuretic	+		*	P
*Verbascum thapsus* L. Scrophulariaceae UNISGVALVER	Couvoùëlp	W	Inflorescences	Infusion Syrup	Respiratory tract inflammations Cough	+	+	***	P
*Verbena officinalis* L. Verbenaceae UNISGWAL032	Barbéno	W	Fever	Infusion	Febrifuge	+		*	P
*Veronica allionii* Vill. Plantaginaceae	See Table 1	W	Flowering aerial parts	Infusion	Diuretic	+		*	C
*Viola calcarata* L. Violaceae UNISGVGB028	Vioulëtto d’mountannho	W	Flowers	Infusion	Respiratory tract inflammations, fever	+	+	**	C
*Viola tricolor* L. Violaceae UNISGVGB005	Vioulëtto blanchoViooulëtin	W	Flowers	Infusion Topically applied	Respiratory tract inflammations, fever, toothache	+	+	***	C
*Unidentified taxon*	Appia	W	Leaves	Topical application	Bruises		+	*	P
*Unidentified taxon*	Murtalia	W	Flowers	Tea	Anti-infllammatory	+		*	P

#: see Table 1 for other voucher codes.

Status: C: cultivated; SC: semi-cultivated or semi-wild; W: wild.

Wal: use recorded among Waldensians; Cat: use recorded among Catholics.

Citations: *quoted by 10% of the informants or less; **quoted by 11-39% of the informants; ***quoted by 40% of the informants or more.

Notes: C: current use; P: past use.

The most common wild medicinal plant-based remedies, which are used externally, comprise the flowers of *Arnica montana*, the aerial parts of *Artemisia absinthium*, the resin of *Abies alba*, and the fresh latex of *Chelidonium majus*. Apart from the last species, this finding confirms the recent ethnobotanical data gathered from other Italian Alpine areas [17-22].

Among the less commonly reported species, the use of the fern *Botrychium lunaria* for skin problems should be further investigated, as the use of this plant was not recorded in the Italian ethnobotanical database compiled in 2004 [38], and the phytochemistry and pharmacology of the genus *Botrychium* is largely unknown, if we exclude the recent work on its flavonoids [39].

The most frequently mentioned local herbal infusions are instead prepared with plants that are commonly used throughout Italy and Europe: *Equisetum arvense, Hypericum perforatum, Parietaria officinalis, Malva sylvestris, Matriciaria chamomilla, Thymus serpyllum, Tilia cordata, Viola tricolor,* and *Cetraria islandica.* The use of the last species is peculiar, however, as it is frequently found, in Italy, in the herbalism-based standardized phytotherapy, but not often in the local folk medical systems.

The remarkable tradition of gathering and using this wild lichen in Waldensian valleys may be, once again, the result of the historical ties that these communities retained with Central and Northern European customs.

The same lichen, gathered from the wild, is also nowadays one of the pillars of the resurgence of the traditional Waldensian cuisine, where it is sometimes used to prepare desserts in a few of the new restaurants in the area [40].

Finally, it is worth mentioning that the unsual herbal folk uses of *Cetraria islandica* and *Botrychium lunaria* find parallelisms in the Alpine Catalan ethnobotany [41,42], showing in this way interesting commonalities between the Catalan and Occitan ethnobotanies of the Alpine communities.

### Veterinary plants

Nearly all the plants pertaining to the veterinary domain (plants used for both feeding and for curing animals, Table 3) were used primarily in the past, as current uses are sporadic and quotation indexes are very low.

**Table 3:** **Local veterinary plant uses recorded in the studied area**

**Botanical taxon/family and voucher specimen code #**	**Local name (folk taxon/generic)**	**Status**	**Plant part(s)**	**Preparation and administration**	**Folk veterinary use(s) or treated desease(s)**	**Treated animals**	**Wal**	**Cat**	**Citations**	**Notes**
*Achillea erba-rotta* All. Asteraceae	See Table 1	W	Aerial parts	Infusion	Rumination disorders	CA	+	+	*	P
*Aconitum napellus* L. Ranunculaceae	Èrbo toro	W	Whole plant	Eaten fresh	Abortive	CA	+		*	P
*Alcea rosea* L. Malvaceae	Malvone	C	Aerial parts	Infusion	Rumination disorders	CA	+		*	P
*Artemisia absinthium* L. Asteraceae	See Table 2	W	Aerial parts	Fodder or in infusions	Rumination disorders	CA, RA	+	+	**	P
*Avena sativa* L. Poaceae	Avéno	C	Aerial parts	Fodder (fresh)	Post-partum depurative	CA		+	*	P
*Calendula officinalis* L*.* Asteraceae	Courtëzio	C	Flowers	Infusion	To facilitate pregnancy	CA	+		*	P
*Cetraria islandica* (L.) Ach. Parmeliaceae	See Table 2	W	Thallus	Decoction	Stomach disorders	CA	+		*	P
*Equisetum arvense* L. Equisetaceae	See Table 2	W	Aerial parts	Foothbath	Infections of the paws	SH		+	*	P
*Euphorbia cyparissias* L. Euphorbiaceae UNISGVGB009	Laitin gró’d mialàourë	W	Fruits	Fodder (dried)	Infections (esp. in the oral cavity)	CA, PO, SH		+	*	P
*Fagopyrum esculentum* Moench. Polygonaceae	Granét	C	Aerial parts	Dried	Fodder	CA, PO, PI	+		*	P
*Festuca ovina* L. Poaceae	Grasoun	W	Aerial parts	Dried	Fodder	CA	+		*	P
*Foeniculum vulgare* Mill. Apiaceae UNISGVGB012	Fënoulh	W	Aerial parts	Fodder (fresh)	Galactagogue	CA	+		*	P
*Fraxinus excelsior* L. Oleaceae	See Table 2	W	Leaves	Fresh	Fodder	CA	+		*	P
*Galium verum* L. Rubiaceae UNISGVALGAL	Caglio	W	Flowering tops	Dried	As rennet		+		*	P
*Gentiana lutea* L. Gentianaceae	See Table 1	W	Roots	Decoction	Rumination disorders	CA, SH	+		*	P
*Heracleum sphondylium* L. Apiaceae	Plaoutasino	W	Aerial parts	Fresh or dried	Fodder	PO, RA	+		*	P
*Juniperus communis* L. Cupressaceae	See Table 1	W	Fruits	Fodder	To improve the skin health (making it shiny)	CA	+		*	P
*Laburnum alpinum* (Mill.) Bercht. & J.Presl. Fabaceae UNISGVGB037	Albuorn	W	Leaves	Fresh or dried	Fodder	RA	+		*	P
*Lamium album* L. Lamiaceae	See Table 2	W	Leaves	Fresh or dried	Fodder	PI, PO, RA	+		*	P
*Larix decidua* Mill. Pinaceae	See Table 2	W	Resin	Topically applied	Bruises, sprains, wounds	CA	+	+	*	C
*Linum usitatissimum* L. Linaceae	See Table 2	C	Seeds	Fodder	“Blood cleansing”	CA		+	*	P
*Malva sylvestris* L. Malvaceae	See Table 2	W	Whole plant	Decoction	Depurative during the menstrual cycle	CA	+		*	P
*Marrubium vulgare* L. Lamiaceae	See Table 2	W	Whole plant	Infusion	Rumination disorders	CA	+		*	P
*Matricaria chamomilla* L*.* Asteraceae	See Table 2	C	Flowers	Infusion	Rumination disorders	CA (calves)	+		*	P
*Medicago sativa* L. Fabaceae	Luzèrno	W	Aerial parts	Fresh or dried	Fodder	CA	+	+	**	C
*Onobrychis viciifolia* Scop. Fabaceae	Jalét	W	Aerial parts	Fresh or dried	Fodder	CA		+	*	P
*Ononis spinosa* L. Fabaceae	See Table 2	W	Roots	Decoction	Depurative during the menstrual cycle	CA	+		*	P
*Oxalis acetosella* L. Oxalidaceae	See Table 1	W	Leaves	Eaten fresh or dry	Fodder	PO, RA	+		*	P
*Parietaria officinalis* L. Urticaceae	See Table 1	C	Aerial parts	Fresh	Fodder	PO	+		*	C
*Pilosella officinarum* Vaill. Asteraceae UNISGVGB013	Èrbo dâ runh	W	Whole plant	Fodder	Rumination disorders	CA	+		*	P
*Plantago major* L.*, P. lanceolata* L. Plantaginaceae	See Table 2	W	Leaves	Fresh or dried	Fodder	PI	+		*	P
*Polyporus officinalis* Fries. Poliporaceae	Panouflo	W	Fruiting body	Fodder (ground)	Rumination disorders	CA	+	+	**	P
*Quercus petraea* (Matt.) Liebl. Fagaceae	Roure	W	Leaves	Fresh or dried	Fodder	GO	+		*	P
*Secale cereale* L. Poaceae	Sèel	C	Seeds→Flour	Fodder	Galactagogue	CA	+	+	*	P
*Sedum album* L. Crassulaceae	Picouloump	W	Leaves	Fresh	Fodder	PO	+		*	P
*Silene vulgaris* (Moench) Garcke Caryophyllaceae UNISGVGB020	Eicloupèt	W	Leaves	Fresh or dried	Fodder	PO, RA	+		*	P
*Stellaria media* (L.) Vill. Caryophyllaceae	Pavarino	W	Leaves	Fresh	Fodder	PO	+		*	P
*Silybum marianum* (L.) Gaertn Asteraceae	Pugn	W	Roots	Decoction	Depurative during the menstrual cycle	CA	+		*	P
*Tanacetum vulgare* L. Asteraceae	See Table 1	W	Aerial parts	Infusion	Rumination disorders	CA		+	*	P
*Taraxacum officinale* F.H.Wigg. Asteraceae	See Table 1	W	Aerial parts	Fresh or dried	Fodder	PO	+		*	P
*Thymus serpyllum* L. Lamiaceae	See Table 1	W	Aerial parts	Topically applied in the mouth	Rumination disorders, infections of the oral cavity	CA, SH	+	+	*	P
*Trifolium alpinum* L. Fabaceae	Fioun	W	Aerial parts	Fresh or dried	Fodder	CA		+	*	P
*Triticum vulgare* Vill. Poaceae	Froumént	C	Aerial parts	Fresh or dried	Fodder	CA	+	+	*	P
*Ulmus glabra* Huds. Ulmaceae UNISGVALULM	Oùëlme	W	Leaves	Fresh or dried	Fodder	PI	+		*	P
*Urtica dioica* L. Urticaceae	See Table 1	W	Leaves	Fresh or dried	Fodder	PO	+		*	P
*Verbascum thapsus* L. Scrophulariaceae	See Table 2	W	Leaves	Fresh or dried	Fodder	SH		+	*	P
*Viola tricolor* L. Violaceae	See Table 1	W	Flowers	Infusion	Rumination disorders	CA	+		*	P

#: see Table 1 and Table 2 for other voucher codes.

Status: C: cultivated; SC: semi-cultivated or semi-wild; W: wild.

Treated animals: CA: cattle; GO: goats; PI: pigs; PO: poultry; RA: rabbits; SH: sheep.

Wal: use recorded among Waldensians; Cat: use recorded among Catholics.

Notes: C: current use; P: past use.

Citations: *quoted by 10% of the informants or less; **quoted by 11% of the informants or more.

This suggests that the socio-economic shift local communities have faced since the 1960s, in which most inhabitants have abandoned the traditional agro-pastoral activities and animal breeding has decreased, has also produced a dramatic loss of Traditional Knowledge concerning veterinary practices.

### Waldensian versus Catholic ethnobotany: the possible role of cultural isolation from neighbours

**Figure 4:**
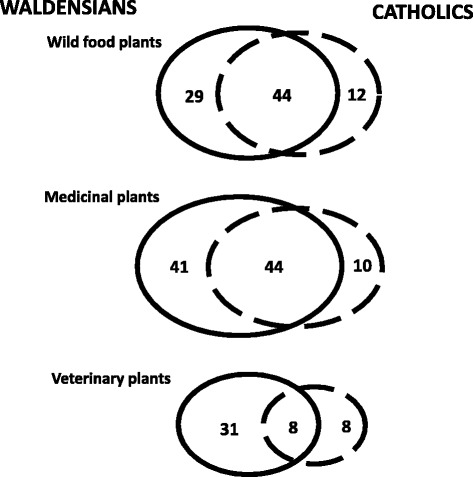
Overlap between the folk plant taxa used among Waldensians and Catholics in the study area.

Figure 4 illustrates the overlap between the ethnobotany of Waldensians and that of their Catholic neighbours in the three analysed domains (folk wild plant foods, medicines, and veterinary food plants and remedies).

The comparison shows that Waldensians had, or have retained, a more extensive ethnobotanical knowledge, and that approximately only half of the recorded wild food and medicinal plants are known and used by both communities. Moreover, this convergence is more marked for the wild food plant domain.

Despite the fact that Waldensians nowadays live together with Catholics, intermarriage between the two religious communities did not exist until a few decades ago. Given the fact that vertical transmission (from grandmother to mothers and from mothers to daughters) of ethnobotanical knowledge is related to kinship networks and these are determined by religious affiliation, this factor may explain the divergence of the two ethnobotanies.

Moreover, the fact that the plant knowledge among Waldensians appears to be more extensive than among the Catholic population may be related to a less marked erosion of the traditional customs and the strong sense of identity Waldensians retain. The historical isolation of the Waldensian community, which survived for many centuries cut off from the rest of their neighbours but at the same time fostered strong ties to Central and Northern Europe, may have facilitated unique patterns of plant perception and use.

However, in the last few decades intermarriage between members of the two communities has become more common (generally bringing the new family into the Catholic faith), and this will probably further hybridize the ethnobotany of the two groups.

On the other hand, a stronger overlap of the ethnobotanies of two culturally distinct groups in the specific wild food domain has also been observed in other mountainous regions of Europe, and may be regarded as a common strategy for coping with the food security-centred struggles that marginalised Alpine populations had to face in the past [1].

### The Waldensian ethnobotany during the last century: a historical analysis

Table 4 illustrates the overlap of ethnobotanical data collected at the end of the 19^th^ Century and in the 1980s in one of the study valleys (Germanasca Valley) [28-30] with our current data.

**Table 4:** **Comparison of the local plant uses recorded in the Germanasca Valley in 1900 and 1984 with those collected in the current study**

**Botanical taxon and family**	**Local uses recorded in 1900** **[** 27 **,** 28 **]**	**Local uses recorded in 1984** **[** 29 **]** *	**Local uses nowadays (current study)**
*Allium cepa* L. (Amaryllidaceae)	NR	Decoction of the bulbs a diuretic	NR
*Amelanchier ovalis* Medik. (Rosaceae)	Fruits consumed as a snack by boys	NR	=
*Anemone hepatica* L. (Ranuncolaceae)	Leaves externally applied on women breast for treating inflammations	NR	NR
*Arctium lappa* L. (Asteraceae)	NR	Infusion of the dried roots, as a depurative	≈
*Arnica montana* L. (Asteraceae)		Alcoholic macerate of the flowers externally applied for treating cuts, rheumatism, and muscle pains	≈
*Artemisia genipi* Weber ex Stechm. (Asteraceae)	NR	Aerial parts in infusion or alcoholic macerate (liquor) as a digestive	=
*Beckwithia glacialis* (L.) Á. Löve & D. Löve (Ranuncolaceae)	Flowers in decoction, drunk as a diaphoretic	Decoction for treating toothaches	NR
*Calendula officinalis* L. (Asteraceae)	NR	Infusion of the dried flowers as a depurative	≈
*Campanula spicata* L. (Campanulaceae)	NR	Fresh leaves, crashed, externally applied for treating cuts	NR
*Cetraria islandica* (L.) Ach. (Parmeliaceae)	NR	Decoction of the thallus as a digestive and expectorant	=
*Chelidonius majus* L. (Papaveraceae)	Latex externally applied on warts	NR	=
*Crataegus rhipidophylla* Gand. (Rosaceae)	Fruits consumed	NR	≠
*Gentiana acaulis* L. (Gentianaceae)	NR	Whole plant or roots in infusion/decoction or wine macerate as appetizing and digestive	=
*Hypericum perforatum* L. (Hypericaceae)	Hung behind the house door, to prevent witcheries	Oil macerate of the fresh flowers as a cicatrizing	= (as in 1984)
*Laburnum anagyroides* Medik. (Fabaceae)	Bark decocted and externally used for treating lice in cows and calves	NR	≠
*Laricifomes officinalis* (Vill.) Kotl. & Pouzar (Fomitopsidaceae)	NR	The fruiting body, powdered, in infusion as a digestive	NR
*Lathyrus sylvestris* (Fabaceae)	Remedy (?) for cows when they calve	NR	NR
*Lilium candidum* L. (Liliaceae)	NR	Oil macerate of the fresh flowers as a cicatrizing	NR
*Linum usitatissimum* L. (Linaceae)	The seeds (in compresses?) as anti-rheumatic	NR	=
*Malva sylvestris* L. (Malvaceae)	Infusion of the leaves (?) as emollient, both for humans and animals	NR	≈
*Nasturtium officinale* R.Br. (Brassicaceae)	Leaves consumed raw in salads	Leaves consumed raw in salads or in soup, as a depurative	NR
*Onobrychis viciifolia* Scop. (Fabaceae)	Fodder	NR	=
*Oxalis acetosella* L. (Oxalidaceae)	Leaves consumed raw in salads	NR	=
*Papaver rhoes* L. (Papaveraceae)	Flowers in decoction, drunk for treating toothache	NR	NR
*Parietaria officinalis* L. (Urticaceae)	NR	Decoction of the dried aerial parts, as a diuretic and depurative	≈
*Polygonum aviculare* L. (Polygonaceae)	NR	Infusion of the dried aerial parts (?) as an astringent	NR
*Rosa canina* L. (Rosaceae)	Flowers consumed as a snack by boys	Infusion of the flowers externally applied for treating eye inflammations	= (as in 1984)
*Rosa centifolia* L. (Rosaceae)	Petals (not clarified how) for treating eye inflammations	NR	≠
*Rubus ideaus* L. (Rosaceae)	Fruits consumed; leaves as fodder	NR	=
*Rubus ulmifolius* Schott (Rosaceae)	Fruits consumed	NR	=
*Sorbus aria* (L.) Crantz (Rosaceae)	Fruits consumed as a snack by boys	NR	≠
*Tanacetum vulgare* L. (Asteraceae)	NR	Fresh aerial parts consumed in salads as a depurative	≈
*Thymus serpyllum* L. (Lamiaceae)	NR	Infusion of the flowering tops as a digestive and anti-tussive	≈
*Tilia x europea* L. (Malvaceae)	Flowers in diaphoretic decoctions; leaves as fodder	NR	=
*Trifolium* spp. (Fabaceae)	Fodder	NR	≈
*Tussilago farfara* L. (Asteraceae)	NR	Crashed fresh leaves, externally applied, as a suppurative	≠
*Urtica dioica* L. (Urticaceae)	NR	Young aerial parts consumed in soups as a depurative; dried roots and leaves, decocted, for treating alopecia; dried leaves used as fodder for hens for increasing the egg production	≈
*Verbascum phlomoides* L. (Scrophulariaceae)	NR	Decoction of the flowers for treating catarrhs	=
*Verbena officinalis* L. (Verbenaceae)	NR	Fresh aerial parts, crashed and mixed with pork fat, externally applied for treating cuts	≠
*Veronica prostrata* L. (Plantaginaceae)	NR	Infusion for treating catarrhs and inflammations	≈
*Viola biflora* L. (Violaceae)	NR	Infusion of the dried flowers for treating coughs and as an intestinal anti-inflammatory; mixed with milk and bread, externally applied, as a suppurative	NR
*Viola calcarata* L. (Violaceae)	Leaves consumed in soups	Infusion of the dried flowers for treating coughs and as an intestinal anti-inflammatory; mixed with milk and bread, externally applied, as a suppurative	= (as in 1984)
*Viola tricolor* L. (Violaceae)	Not specified, the resulting preparation (decoction of the aerial parts?) considered good for those women, who had given a baby	Infusion of the dried flowers for treating coughs and as an intestinal anti-inflammatory; mixed with milk and bread, externally applied, as a suppurative	≠

*We considered folk uses referred only to those plant taxa, for which local names were reported.

(?): hypothesized plant use details.

NR: not recorded; = same use; ≈ similar use; ≠ different uses.

Although few plants were reported in the ethnobotanical study published in 1900 [28,29] and few taxa were reported with their local names in the survey published in 1984 [30] (thus suggesting maybe a sampling based mainly on trained herbalists), more than half of these species recorded in these two studies are used in the same of similar ways today.

However, possible different research methods used in the current and past field studies make a detailed comparison very problematic, as in both of the past considered surveys, which were conducted by botanists, an exact description of the utilized sampling and ethnographic methods and, paradoxically, even an indication of collected plant vouchers are completely missing.

The comparative analysis shows in any case a remarkable degree of resilience of traditional plant uses in the study area, despite the tremendous socio-economic changes that occurred during the last 120 years; other diachronic analyses recently conducted in the Balkans have also confirmed the survival of 19^th^ Century folk plant uses to today [16,43].

## Conclusions

Local plants have played, and still partially play, an important role in the context of food security and emic, domestic pathways of the management of human and animal health in the Western Alps.

A marked persistence of local knowledge regarding these plants among Waldensians confirms the importance of studying enclaves as well as cultural and linguistic “isles” in ethnobotany, which may represent both crucial reservoirs of folk knowledge and *bio-cultural refugia* [44].

On the other hand, the findings of this study indicate that a proper conservation of the bio-cultural heritage, such as the ethnobotanical one, requires strategies, which carefully consider natural landscapes and resources as well as cultural and religious customs, since plant folk knowledge systems are the result of a continuous interplay between these two domains over centuries.

Finally, these neglected local plant resources may represent a key issue for fostering a sustainable development in an area of the Alps, which has been largely untouched by mass tourism and is looking with particular interest at eco-touristic trajectories.
